# Medicaid Expansion and Restriction Policies for Hepatitis C Treatment

**DOI:** 10.1001/jamanetworkopen.2024.22406

**Published:** 2024-07-16

**Authors:** Nathan W. Furukawa, Susan Z. Ingber, Hasan Symum, Karina K. Rapposelli, Eyasu H. Teshale, William W. Thompson, Weiming Zhu, Henry W. Roberts, Neil Gupta

**Affiliations:** 1 Division of Viral Hepatitis, National Center for HIV, Viral Hepatitis, STD, and TB Prevention, Centers for Disease Control and Prevention, Atlanta, Georgia; 2 Division of HIV Prevention, National Center for HIV, Viral Hepatitis, STD, and TB Prevention, Centers for Disease Control and Prevention, Atlanta, Georgia

## Abstract

**Importance:**

Hepatitis C can be cured with direct-acting antivirals (DAAs), but Medicaid programs have implemented fibrosis, sobriety, and prescriber restrictions to control costs. Although restrictions are easing, understanding their association with hepatitis C treatment rates is crucial to inform policies that increase access to lifesaving treatment.

**Objective:**

To estimate the association of jurisdictional (50 states and Washington, DC) DAA restrictions and Medicaid expansion with the number of Medicaid recipients with filled prescriptions for DAAs.

**Design, Setting, and Participants:**

This cross-sectional study used publicly available Medicaid documents and claims data from January 1, 2014, to December 31, 2021, to compare the number of unique Medicaid recipients treated with DAAs in each jurisdiction year with Medicaid expansion status and categories of fibrosis, sobriety, and prescriber restrictions. Medicaid recipients from all 50 states and Washington, DC, during the study period were included. Multilevel Poisson regression was used to estimate the association between Medicaid expansion and DAA restrictive policies on jurisdictional Medicaid DAA prescription fills. Data were analyzed initially from August 15 to November 15, 2023, and subsequently from April 15 to May 9, 2024.

**Exposures:**

Jurisdictional Medicaid expansion status and fibrosis, sobriety, and prescriber DAA restrictions.

**Main Outcomes and Measures:**

Number of people treated with DAAs per 100 000 Medicaid recipients per year.

**Results:**

A total of 381 373 Medicaid recipients filled DAA prescriptions during the study period (57.3% aged 45-64 years; 58.7% men; 15.2% non-Hispanic Black and 52.2% non-Hispanic White). Medicaid nonexpansion jurisdictions had fewer filled DAA prescriptions per 100 000 Medicaid recipients per year than expansion jurisdictions (38.6 vs 86.6; adjusted relative risk [ARR], 0.56 [95% CI, 0.52-0.61]). Jurisdictions with F3 to F4 (34.0 per 100 000 Medicaid recipients per year; ARR, 0.39 [95% CI, 0.37-0.66]) or F1 to F2 fibrosis restrictions (61.9 per 100 000 Medicaid recipients per year; ARR, 0.62 [95% CI, 0.59-0.66]) had lower treatment rates than jurisdictions without fibrosis restrictions (94.8 per 100 000 Medicaid recipients per year). Compared with no sobriety restrictions (113.5 per 100 000 Medicaid recipients per year), 6 to 12 months of sobriety (38.3 per 100 000 Medicaid recipients per year; ARR, 0.65 [95% CI, 0.61-0.71]) and screening and counseling requirements (84.7 per 100 000 Medicaid recipients per year; ARR, 0.87 [95% CI, 0.83-0.92]) were associated with reduced treatment rates, while 1 to 5 months of sobriety was not statistically significantly different. Compared with no prescriber restrictions (97.8 per 100 000 Medicaid recipients per year), specialist consult restrictions was associated with increased treatment (66.2 per 100 000 Medicaid recipients per year; ARR, 1.05 [95% CI, 1.00-1.10]), while specialist required restrictions were not statistically significant.

**Conclusions and Relevance:**

In this cross-sectional study, Medicaid nonexpansion status, fibrosis, and sobriety restrictions were associated with a reduction in the number of people with Medicaid who were treated for hepatitis C. Removing DAA restrictions might facilitate treatment of more people diagnosed with hepatitis C.

## Key Points

**Question:**

To what extent have Medicaid expansion status and prior authorization requirements limited access to hepatitis C direct-acting antivirals (DAAs) among Medicaid recipients?

**Findings:**

In this cross-sectional analysis of 381 373 Medicaid recipients who filled DAA prescriptions from 2014 to 2021, Medicaid nonexpansion status, fibrosis restrictions, and sobriety restrictions were all associated with a reduction in hepatitis C treatment rates among Medicaid recipients.

**Meaning:**

These findings suggest that removing prior authorization requirements will increase hepatitis C treatment rates.

## Teaser

This cross-sectional study assesses the association of Medicaid expansion and restrictions on prescription of direct-acting antivirals with the rate of Medicaid recipients treated for hepatitis C nationwide.

## Introduction

Since 2014, use of oral direct-acting antivirals (DAAs) has been the standard of care for hepatitis C and results in cure rates of greater than 95% with 8 to 12 weeks of treatment.^1^ Although the US has a goal of eliminating hepatitis C as a public health threat by 2030, the number of estimated new hepatitis C virus (HCV) infections has more than doubled from 24 700 in 2012 to 69 800 in 2021.^2,3^ After an initial surge of people being treated after the release of DAAs, the number of people treated for hepatitis C has steadily declined from a peak of 164 247 people in 2015 to 83 740 people in 2020.^4^ Previous modeling has suggested that a mean of 260 000 people must be treated each year to reach hepatitis C elimination targets.^5^ Despite an estimate of more than 1 million people treated with DAAs in the US since 2014, only 34% of people with diagnosed infection had evidence of viral clearance, and the number of people with hepatitis C nationally remained stable from 2.1 million in 2013 to 2016 to 2.2 million in 2017 to 2020.^6,7^ Many more people with hepatitis C need to be treated to reach national hepatitis C elimination targets.

Among the estimated 2.2 million people with hepatitis C, 60% have public insurance, defined as Medicare, Medicaid, or other government health plan, and 44% have incomes below the poverty line.^7^ Medicaid programs play a key role in insuring adult populations who are not covered by commercial insurance or Medicare. Although Medicaid programs receive a rebate off the average manufacturer price, the high initial DAA cost of $84 000 to $94 500 per treatment regimen prohibited programs from treating all Medicaid recipients with hepatitis C.^8^ To manage costs, Medicaid programs implemented DAA restrictions based on stages of liver fibrosis, requirements for sobriety, and prescribers’ specialties.^9^ Removal of all 3 DAA restrictions as well as removing the need for the prescriber to submit additional information prior to approval of the DAA prescription is considered prior authorization removal. Although the national average drug acquisition cost of DAAs has fallen to as low as $26 000 per treatment regimen and Medicaid programs have gradually relaxed treatment restrictions, many programs still have restrictions in place that limit access to treatment.^9,10^ As a result, only 31% of people with Medicaid and evidence of HCV infection have evidence of viral clearance, compared with 40% of people with commercial insurance and 45% of people with Medicare.^6^ Further, only 23% of people with Medicaid are treated within 1 year of HCV infection diagnosis, compared with 28% of Medicare recipients and 35% of commercial insurance recipients.^11^

Previous studies have demonstrated a relative increase in DAA prescriptions following the lifting of DAA restrictions.^12,13,14,15^ However, an assessment of the overall association between Medicaid expansion and these restrictions with the rate of people with Medicaid treated for hepatitis C nationwide may inform decisions to remove persistent prior authorization requirement. Furthermore, the historical experience of implementing DAA prior authorization requirements to prioritize hepatitis C treatment among certain groups may inform future decisions around implementing prior authorization for treatment of other conditions with public health implications.

## Methods

This cross-sectional study was approved by the National Center for HIV, Viral Hepatitis, STD, and TB Prevention at the Centers for Disease Control and Prevention. Because the work was deemed to not be human subjects research, the need for informed consent was waived. The study followed the Strengthening the Reporting of Observational Studies in Epidemiology (STROBE) reporting guideline.

### People With Medicaid Prescribed DAAs

The Centers for Medicare & Medicaid Services (CMS) captures prescription data from all plan types supported by Medicaid. The CMS Transformed Medicaid Statistical Information System Analytic Files and Medicaid Analytic eXtract databases were used to identify individuals who filled DAA prescriptions from January 1, 2014, to December 31, 2021. For each Medicaid beneficiary, prescription information was longitudinally linked across these 2 databases using a unique identification provided by the CMS. Initiation of DAA treatment was defined as the filling of any prescription drug corresponding to an HCV DAA using the US Food and Drug Administration’s National Drug Codes definition (eTable 1 in Supplement 1). The number of people who had their first DAA prescription filled in each jurisdiction (50 states and Washington, DC) were counted from 2014 to 2021. The analysis was restricted to the date of first DAA fill and did not account for subsequent courses of treatment relating to nonadherence, treatment failure, or HCV reinfection. Therefore, patients were counted only once in the analysis and were assigned to the year of their first treatment. Jurisdictions with fewer than 10 prescriptions in a given year were censored.

The mean number of monthly Medicaid recipients for each jurisdiction was used to estimate a mean annual Medicaid census for each jurisdiction-year.^16^ The number of people with Medicaid who filled their first DAA prescriptions in each jurisdiction-year was divided by the mean Medicaid census for each jurisdiction during that year to generate the number of people with filled DAA prescriptions per 100 000 Medicaid recipients per year.

### Medicaid Expansion and DAA Restrictions

The date of Medicaid expansion was determined for each jurisdiction and year using data from the Kaiser Family Foundation.^17^ Medicaid was considered expanded for the given jurisdiction-year if Medicaid expansion implementation occurred at any point in the calendar year. Longitudinal data on jurisdictional Medicaid DAA restriction policy status and lift dates were collected from May 1, 2021, to January 31, 2022, through a search and triangulation of multiple publicly available records. Sources of 2014 to 2021 policy data included publications,^18^ the National Viral Hepatitis Round Table and Harvard Center for Health Law and Policy Innovation State of Hepatitis C reports,^9,19^ and jurisdictional Medicaid documents. The earliest DAA policy restriction change that happened in the calendar year was applied to the jurisdiction year in which the policy change happened (ie, the earliest date when ≥1 Medicaid plan in a jurisdiction adopted a restriction reduction or lift).

Fibrosis restrictions were categorized into none, requiring F1 (mild) to F2 (moderate) fibrosis, or requiring F3 (severe) to F4 (cirrhosis) fibrosis. Sobriety restrictions were categorized into none, screening or counseling for substance use disorder (SUD), 1 to 5 months of sobriety, or 6 to 12 months of sobriety. Prescriber restrictions were categorized into none, specialist consult (requiring the clinician to consult a specialist, but allowing DAAs to be prescribed by any clinician), or specialist required (only clinicians such as infectious disease or gastroenterology specialists could prescribe DAAs). Jurisdiction years where the DAA restriction policies were not available were counted as unknown (eTable 2 in Supplement 1).

### Statistical Analysis

Data were analyzed initially from August 15 to November 15, 2023, and subsequently from April 15 to May 9, 2024. The number of people with Medicaid coverage who filled DAA prescriptions, the number of people with Medicaid coverage, Medicaid expansion status, and DAA restrictive policies were compared for each year from 2014 to 2021. The mean annual number of people with filled DAA prescriptions per 100 000 Medicaid recipients per year in each jurisdiction was then compared by year, Medicaid expansion status, and DAA restrictive policies. Multilevel Poisson regression was used to measure the association of Medicaid expansion status and fibrosis, sobriety, and prescriber restrictions on the number of people with filled DAA prescriptions per Medicaid population. Random effects for jurisdiction and year were included in the model to adjust for correlated observations. Analysis was performed using SAS, version 9.4 (SAS Institute Inc). Two-sided *P* < .05 indicated statistical significance.

## Results

A total of 381 373 people with Medicaid had filled DAA prescriptions from 2014 to 2021, increasing from 20 516 in 2014 to a peak of 64 974 in 2019 and decreasing to 53 708 in 2021 (Table 1). Only 4 jurisdiction years (<1%) were excluded due to fewer than 10 prescriptions occurring that year (Alaska in 2014, North Dakota in 2015 and 2017, and South Dakota in 2016). Among the 381 373 Medicaid recipients filling DAA prescriptions, 218 479 (57.3%) were aged 45 to 64 years, 223 804 (58.7%) were men, 151 224 (39.7%) were women, and 6345 (1.7%) had data on sex missing. Among those with race and ethnicity information available, 6148 recipients (1.6%) were Asian; 39 632 (10.4%) were Hispanic, 58 023 (15.2%) were non-Hispanic Black, 199 159 (52.2%) were non-Hispanic White, and 6757 (1.8%) were of other race or ethnicity (including American Indian or Alaska Native and multiracial). Most recipients (119 206 [31.3%]) resided in the Northeast (eTable 3 in Supplement 1).

**Table 1.** Study Measures by Study Year

Measure	Study year							
	2014	2015	2016	2017	2018	2019	2020	2021
Total No. of people who filled DAA prescriptions	20 516	35 981	44 829	49 628	57 529	64 974	54 208	53 708
Total mean Medicaid population, millions	67.4	74.5	74.6	74.1	74.8	73.6	77.6	76.7
No. of jurisdictions with Medicaid expansion								
Yes	27	30	32	32	32	34	37	38
No	24	21	19	19	19	17	14	13
No. of jurisdictions with fibrosis restrictions								
None	2	3	11	21	36	43	46	49
F1-F2	3	4	16	19	8	4	2	1
F3-F4	31	30	21	11	7	4	3	1
Unknown	15	14	3	0	0	0	0	0
No. of jurisdictions with sobriety restrictions								
None	2	4	3	10	13	16	18	24
SUD screening or counseling	9	7	10	15	19	18	20	16
Documentation of abstinence, mo								
1-5	8	8	10	19	8	8	7	5
6-12	21	21	21	7	11	9	6	6
Unknown	11	11	7	0	0	0	0	0
No. of jurisdictions with prescriber restrictions								
None	1	2	3	14	19	22	29	35
Specialist consult^a^	15	16	25	27	28	26	20	15
Specialist required^b^	14	10	10	9	4	3	2	1
Unknown	21	23	13	1	0	0	0	0
No. of jurisdictions requiring prior authorization								
Prior authorization removed	0	0	0	0	0	2	5	9
Prior authorization required	51	51	51	51	51	49	46	42

Abbreviations: DAA, direct-acting antiviral; F1, mild fibrosis; F2, moderate fibrosis; F3, severe fibrosis; F4, cirrhosis; SUD, substance use disorder.

^a^ Required consultation from a specialist, usually infectious diseases, gastroenterology, or hepatology, but any clinician could prescribe DAAs.

^b^ Restricted DAA prescribing to only specialists.

The number of jurisdictions that expanded Medicaid increased from 27 in 2014 to 38 in 2021. Among jurisdictions with known DAA restriction policies in 2014, 34 of 36 (94.4%) had a fibrosis restriction, 38 of 40 (95.0%) had a sobriety restriction, and 29 of 30 (96.7%) had a prescriber restriction in place. Restrictions were gradually lifted over time and by 2021, only 2 of 51 jurisdictions (3.9%) had a fibrosis restriction, 27 of 51 (53.0%) had a sobriety restriction, and 16 of 51 (31.4%) had a prescriber restriction. By 2021, 9 jurisdictions had fully removed prior authorization requirements.

The mean number of people with filled DAA prescriptions per 100 000 Medicaid recipients per year increased from 30.8 (95% CI, 23.6-38.0) in 2014 to a peak of 99.0 (95% CI, 80.0-118.0) in 2019 before declining to 81.8 (95% CI, 66.5-97.0) in 2021 (Table 2). Jurisdictions that did not expand Medicaid had fewer people with filled DAA prescriptions per 100 000 Medicaid recipients per year compared with jurisdictions that did expand Medicaid (38.6 [95% CI, 34.4-42.9] vs 86.6 [95% CI, 79.6-93.6]) (Figure). Jurisdictions that restricted DAAs to those with advanced F3 to F4 fibrosis had fewer people with filled DAA prescriptions per 100 000 Medicaid recipients per year (34.0 [95% CI, 29.4-38.5]) than jurisdictions with F1 to F2 fibrosis restrictions (61.9 [95% CI, 51.6-72.2]) or no restrictions (94.8 [95% CI, 86.9-102.6]). Jurisdictions with strict 6 to 12 months of sobriety restrictions had fewer people with filled DAA prescriptions per 100 000 Medicaid recipients per year (38.3 [95% CI, 33.0-43.6]) compared with jurisdictions requiring 1 to 5 months of sobriety (47.1 [95% CI, 38.5-55.6]) or SUD screening or counseling (84.7 [95% CI, 74.1-95.2]) and those with no restrictions (113.5 [95% CI, 102.1-124.9]). Jurisdictions where a specialist was required had fewer people who filled DAA prescriptions per 100 000 Medicaid recipients per year (44.9 [95% CI, 35.2-54.6]) compared with jurisdictions where only a specialist consult was needed (66.2 [95% CI, 58.4-74.0]) and jurisdictions without prescriber restrictions (97.8 [95% CI, 87.4-108.3]). Last, the rate of DAA prescriptions filled per 100 000 Medicaid recipients per year was higher among the 16 jurisdictions that removed prior authorization (104.6 [95% CI, 74.7-134.5]) compared with the jurisdictions that still required prior authorization (68.2 [95% CI, 62.8-73.5]). Comparisons of the number of people with filled DAA prescriptions per 100 000 Medicaid recipients per year by Medicaid expansion status and DAA restrictions over time are included in eFigures 1 to 4 in Supplement 1.

**Table 2.** Unadjusted Mean Number of People Who Filled Direct-Acting Antiviral Prescriptions per 100 000 Medicaid Recipients per Year

Stratification	Contributing jurisdiction years	No. treated per 100 000 Medicaid recipients per year (95% CI)
Overall	NA	69.6 (64.3-74.9)
Year		
2014	50	30.8 (23.6-38.0)
2015	50	49.4 (37.8-61.1)
2016	50	57.9 (45.4-70.5)
2017	50	68.9 (56.1-81.6)
2018	51	83.3 (67.8-98.9)
2019	51	99.0 (80.0-118.0)
2020	51	84.4 (68.5-100.2)
2021	51	81.8 (66.5-97.0)
Jurisdictions with Medicaid expansion		
Yes	261	86.6 (79.6-93.6)
No	143	38.6 (34.4-42.9)
Jurisdictions with fibrosis restrictions		
None	210	94.8 (86.9-102.6)
F1-F2	57	61.9 (51.6-72.2)
F3-F4	106	34.0 (29.4-38.5)
Jurisdictions with sobriety restrictions		
None	90	113.5 (102.1-124.9)
SUD screening or counseling	114	84.7 (74.1-95.2)
Documented sobriety, mo		
1-5	60	47.1 (38.5-55.6)
6-12	112	38.3 (33.0-43.6)
Jurisdictions with prescriber restrictions		
None	125	97.8 (87.4-108.3)
Specialist consult^a^	171	66.2 (58.4-74.0)
Specialist required^b^	52	44.9 (35.2-54.6)
Jurisdictions requiring prior authorization		
Prior authorization removed	16	104.6 (74.7-134.5)
Prior authorization required	392	68.2 (62.8-73.5)

Abbreviations: F1, mild fibrosis; F2, moderate fibrosis; F3, severe fibrosis; F4, cirrhosis; NA, not applicable; SUD, substance use disorder.

^a^ Required consultation from a specialist, usually infectious diseases, gastroenterology, or hepatology, but any clinician could prescribe direct-acting antivirals.

^b^ Restricted direct-acting antiviral prescribing to only specialists.

**Figure.**
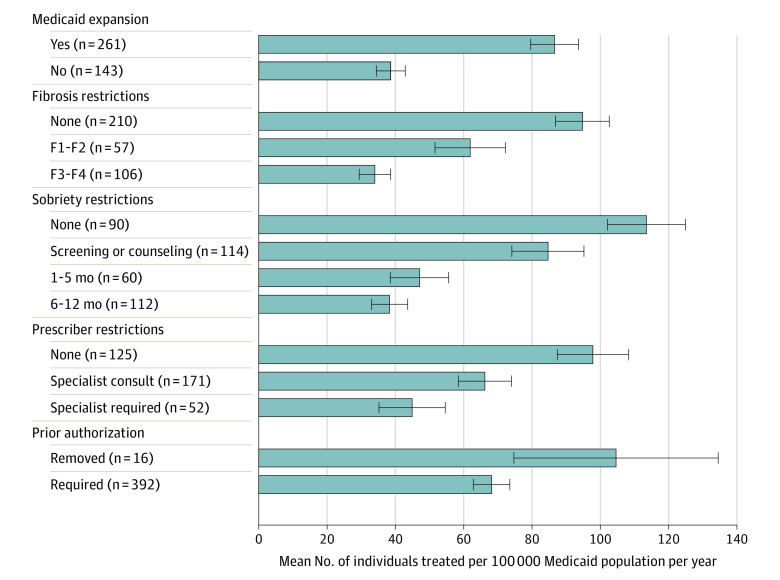
Unadjusted Annual Number of People With Filled Prescriptions for Direct-Acting Antivirals (DAAs) per 100 000 Medicaid Recipients per Year by Jurisdictional Medicaid Expansion Status and DAA Restriction Policies Data are from the US, 2014 to 2021. Numbers in parentheses represent the number of contributing jurisdiction years across the study period. Jurisdictions can contribute discrete jurisdiction years to each category and may contribute to multiple categories across the study period. Restrictions eased over time and the median year for each covariate is 2018 for Medicaid expansion, 2017 for Medicaid nonexpansion, 2019 for no fibrosis restrictions, 2017 for F1 (mild) to F2 (moderate) fibrosis restrictions, 2015 for F3 (severe) to F4 (cirrhosis) fibrosis restrictions, 2019 for no sobriety restrictions, 2018 for substance use disorder screening or counseling, 2015 for sobriety of 1 to 5 months, 2016 for sobriety of 6 to 12 months, 2020 for no prescriber restrictions, 2018 for specialist consultation, 2016 for specialist prescriber, 2021 for prior authorization removed, and 2017 for prior authorization required. Error bars indicate 95% CIs.

In bivariate Poisson regression, Medicaid nonexpansion status and fibrosis, sobriety, and prescriber restrictions were all associated with fewer filled DAA prescriptions per 100 000 Medicaid recipients per year and were included in the multilevel model (Table 3). In the multilevel model, not expanding Medicaid was associated with fewer people with filled DAA prescriptions per 100 000 Medicaid recipients per year (adjusted relative risk [ARR], 0.56 [95% CI, 0.52-0.61]). Compared with jurisdictions without fibrosis restrictions, jurisdictions with F1 to F2 fibrosis restrictions had fewer people with filled DAA prescriptions per 100 000 Medicaid recipients per year (ARR, 0.62 [95% CI, 0.59-0.66]), while jurisdictions with F3 to F4 fibrosis restrictions had even fewer people with filled DAA prescriptions per 100 000 Medicaid recipients per year (ARR, 0.39 [95% CI, 0.37-0.43]). Compared with jurisdictions without sobriety restrictions, jurisdictions with 6 to 12 months of sobriety (ARR, 0.65 [95% CI, 0.61-0.72]) and SUD screening or counseling (ARR, 0.87 [95% CI, 0.83-0.92]) requirements had fewer people with filled DAA prescriptions per 100 000 Medicaid recipients per year, while 1 to 5 months of sobriety requirement did not have a significantly different number of people with filled prescriptions. Finally, compared with jurisdictions without prescriber restrictions, the number of people with filled DAA prescriptions per 100 000 Medicaid recipients per year among jurisdictions with specialist consult restrictions was marginally higher (ARR, 1.05 [95% CI, 1.00-1.10]), while those in jurisdictions with specialist required restrictions were not significantly different. However, based on sensitivity analyses, there was a high degree of collinearity with sobriety and prescriber restrictions such that when both variables were included in the model, the effects were less interpretable (eTable 4 in Supplement 1).

**Table 3.** Bivariate and Multilevel Models of Jurisdictional Medicaid Expansion and Restrictive Policies on People Who Filled Direct-Acting Antiviral Prescriptions per 100 000 Medicaid Recipients per Year

Stratification	Bivariate model		Multilevel model	
	RR (95% CI)	*P* value	ARR (95% CI)	*P* value
Jurisdictions with Medicaid expansion				
Yes	1 [Reference]	NA	1 [Reference]	NA
No	0.45 (0.35-0.57)	<.001	0.56 (0.52-0.61)	<.001
Jurisdictions with fibrosis restrictions				
None	1 [Reference]	NA	1 [Reference]	NA
F1-F2	0.64 (0.49-0.84)	.002	0.62 (0.59-0.66)	<.001
F3-F4	0.34 (0.27-0.42)	<.001	0.39 (0.37-0.43)	<.001
Jurisdictions with sobriety restrictions				
None	1 [Reference]	NA	1 [Reference]	NA
SUD screening or counseling	0.78 (0.61-0.99)	.04	0.87 (0.83-0.92)	<.001
Documented sobriety required, mo				
1-5	0.42 (0.28-0.63)	<.001	1.08 (0.97-1.2)	.14
6-12	0.34 (0.27-0.43)	<.001	0.65 (0.61-0.71	<.001
Jurisdictions with provider restrictions				
None	1 [Reference]	NA	1 [Reference]	NA
Specialist consult^a^	0.68 (0.54-0.87)	.002	1.05 (1.00-1.10)	.05
Specialist required^b^	0.46 (0.32-0.65)	<.001	1.07 (0.99-1.15)	.08
Jurisdictions requiring prior authorization				
Prior authorization removed	1 [Reference]	NA	NA	NA
Prior authorization required	0.71 (0.47-1.07)	.10	NA	NA

Abbreviations: ARR, adjusted relative risk (ARR); F1, mild fibrosis; F2, moderate fibrosis; F3, severe fibrosis; F4, cirrhosis; NA, not applicable; RR, relative risk; SUD, substance use disorder.

^a^ Required consultation from a specialist, usually infectious diseases, gastroenterology, or hepatology, but any clinician could prescribe direct-acting antivirals.

^b^ Restricted direct-acting antiviral prescribing to only specialists.

## Discussion

Medicaid programs plays a crucial role in ensuring health insurance coverage of adults with lower incomes who might otherwise not be able to access hepatitis C treatment. Due to the higher cost of DAAs, restrictive DAA policies were first implemented to prioritize patients with the most advanced liver disease, patients considered most likely to complete treatment, and patients who had a specialist involved in their care. This analysis demonstrates an association between Medicaid nonexpansion status and restrictive DAA policies resulting in fewer people with Medicaid receiving DAA treatment. For instance, our study found that jurisdictions with Medicaid expansion treated almost twice as many people compared with jurisdictions without Medicaid expansion, while jurisdictions with no fibrosis restrictions treated more than twice as many people compared with jurisdictions requiring F3 to F4 fibrosis before treatment. Although Medicaid expansion increases the denominator of Medicaid recipients, the population of younger childless adults with lower incomes covered under Medicaid expansion may be at higher risk for HCV infection and receive treatment at higher rates than the population with preexisting Medicaid coverage. Increased removal of prior authorization requirements was not statistically significant in this analysis, likely owing to the limited number of jurisdictions that had removed prior authorization in the most recent available data for this study. Future analyses on data from 2022 onward may show a further increase in hepatitis C treatment rates due to decreased delays between DAA prescription and dispensing. While there was a marginal increase in hepatitis C treatment associated with specialist consult restrictions in the multilevel model, this result may be spurious due to collinearity with changes in sobriety restrictions and, although statistically significant, the difference was marginal for treatment rates compared with the other variables.

Although designed to reduce short-term costs, many people with hepatitis C have been left behind as a result of these DAA restriction policies, and progress toward hepatitis C elimination has been hindered. Nearly a decade since DAAs were released, the number of people with hepatitis C has been stagnant at more than 2 million.^7^ There are substantial benefits of hepatitis C treatment for patients, public health, and payers. For patients, treatment and cure of hepatitis C confers significantly reduced mortality and morbidity from hepatitis C–related liver disease, chronic kidney disease, cardiovascular disease, and type 2 diabetes.^20,21^ For public health, hepatitis C treatment also has a prevention effect, reducing the potential for HCV transmission as increasingly fewer people engaging in higher-risk behavior such as injection drug use have HCV viremia.^22,23,24^ Last, payers benefit because hepatitis C cure is associated with fewer health care costs from hepatitis C complications; treatment is estimated to be cost-neutral within 3 to 5 years and substantially cost saving over a lifetime.^25,26^

As the price of DAAs has gradually decreased, Medicaid programs have continued to remove barriers to hepatitis C treatment. As of early 2024, no jurisdiction has fibrosis restrictions, 9 maintain an SUD screening or a sobriety requirement, and 4 retain prescriber restrictions.^9^ Additionally, 28 jurisdictions have completely removed all prior authorization requirements. While this represents substantial improvement, the presence of any restriction presents a barrier to care and a missed opportunity for scaling up hepatitis C treatment. Furthermore, although Medicaid DAA restrictions are publicly available, similar prior authorization data from commercial insurers are not available and remain unstudied. Restrictions on DAAs are increasingly difficult to justify in an era where treating people is cost saving,^27^ people who use drugs can achieve cure rates similar to that of the general population,^28,29^ and primary care clinicians can treat most cases of hepatitis C with simplified guidelines.^30^ Removal of DAA restrictions may facilitate treatment of the backlog of Medicaid recipients waiting for hepatitis C treatment and accelerate progress toward elimination of hepatitis C.

### Limitations

This study has several limitations. First, the documentation of DAA restriction policies during 2014 to 2016 was limited, potentially underestimating the association of undocumented restrictions in place at that time with study outcomes. Second, the primary outcome measure uses the number of Medicaid recipients as the denominator rather than the number of Medicaid recipients with current HCV infection. Unfortunately, there is no reliable estimate of hepatitis C prevalence among Medicaid recipients by jurisdiction, and estimates using claims data are inadequate due to the lack of laboratory test results. Third, our analysis assumed that Medicaid policies in a given year applied to all Medicaid-supported plans. Although managed care organization plans are required to be at least as permissive as jurisdiction fee-for-service plans, DAA restrictive policy changes may take several months to years to be reflected in all Medicaid-supported plans, resulting in potential misclassification. However, most policy changes occurred in the first half of the study period, and data from subsequent jurisdiction years dilute the effect of this misclassification. Fourth, a limited number of jurisdictions had completely removed prior authorization requirements to enable definitive conclusions on its independent association relative to specific fibrosis, sobriety, and prescriber restrictions. Last, this ecologic analysis explores the association between Medicaid policies and hepatitis C treatment. Because differences in local testing practices, linkage to care, and treatment capacity might also be associated with jurisdictional Medicaid policies and hepatitis C treatment outcomes, the possibility of residual confounding persists.

## Conclusions

The findings of this cross-sectional analysis suggest that the number of people with Medicaid who are treated for hepatitis C was lower in jurisdictions with Medicaid nonexpansion and fibrosis and sobriety DAA restrictive policies. While a national hepatitis C elimination initiative including a subscription-based payment model covering Medicaid recipients has been proposed,^31^ Medicaid programs currently can remove all restrictive DAA prior authorization policies. This could potentially improve timely access to hepatitis C treatment for thousands of people. Fully removing DAA prior authorization could also reduce disparities in hepatitis C treatment access and enhance health equity among people who use drugs or alcohol, people experiencing poverty, and people without access to specialty care. In the absence of urgent interventions to improve access to lifesaving DAAs, hepatitis C treatment rates may continue to decline and diminish national progress of hepatitis C elimination efforts.

## Notes

Supplement 1. **eTable 1.** Direct-Acting Antiviral National Drug Codes Used for Medicaid Analysis **eTable 2.** Medicaid Expansion and DAA Restrictive Policy Categorization—US, 2014-2021 **eTable 3.** Baseline Characteristics of Individuals With Medicaid Filling HCV DAA Prescriptions—US, 2014-2021 **eTable 4**. Sensitivity Analysis of Multilevel Models of Jurisdictional Medicaid Expansion and DAA Restrictive Policies on DAA Prescription Fill Rates Among Medicaid Recipients Varying Sobriety and Prescriber Restrictions—US, 2014-2021 **eFigure 1.** Unadjusted Rate of DAA Prescriptions Fills per 100 000 Medicaid Recipients per Year by Jurisdictional Medicaid Expansion Status—US, 2014-2021 **eFigure 2.** Unadjusted Rate of DAA Prescriptions Fills per 100 000 Medicaid Recipients per Year by Jurisdictional Fibrosis Restrictions—US, 2014-2021 **eFigure 3.** Unadjusted Rate of DAA Prescriptions Fills per 100 000 Medicaid Recipients per Year by Jurisdictional Sobriety Restrictions—US, 2014-2021 **eFigure 4.** Unadjusted Rate of DAA Prescriptions Fills per 100 000 Medicaid Recipients per Year by Jurisdictional Prescriber Restrictions—United States, 2014-2021

Supplement 2.
Data Sharing Statement

## References

[1] Marks K, Naggie S. Management of hepatitis C in 2019. JAMA. 2019;322(4):355-356. doi:10.1001/jama.2019.5353 31099822

[2] US Department of Health and Human Services. Viral Hepatitis National Strategic Plan overview: what is the Viral Hepatitis National Strategic Plan? Reviewed January 7, 2021. Accessed March 25, 2021. https://www.hhs.gov/hepatitis/viral-hepatitis-national-strategic-plan/national-viral-hepatitis-action-plan-overview/index.html

[3] Centers for Disease Control and Prevention. 2021 Viral Hepatitis Surveillance Report. Reviewed August 7, 2023. Accessed September 23, 2023. https://www.cdc.gov/hepatitis/statistics/2021surveillance/index.htm

[4] Teshale EH, Roberts H, Gupta N, Jiles R. Characteristics of persons treated for hepatitis C using National Pharmacy Claims Data, United States, 2014-2020. Clin Infect Dis. 2022;75(6):1078-1080. doi:10.1093/cid/ciac139 35171997

[5] National Academies of Sciences, Engineering, and Medicine; Health and Medicine Division; Board on Population Health and Public Health Practice; Committee on a National Strategy for the Elimination of Hepatitis B and C. A National Strategy for the Elimination of Hepatitis B and C: Phase Two Report. National Academies Press; 2017. 28737845

[6] Wester C, Osinubi A, Kaufman HW, . Hepatitis C virus clearance cascade—United States, 2013-2022. MMWR Morb Mortal Wkly Rep. 2023;72(26):716-720. doi:10.15585/mmwr.mm7226a3 37384551 PMC10328490

[7] Lewis KC, Barker LK, Jiles RB, Gupta N. Estimated prevalence and awareness of hepatitis C virus infection among US adults—National Health and Nutrition Examination Survey, January 2017–March 2020. Clin Infect Dis. 2023;77(10):1413-1415. doi:10.1093/cid/ciad411 37417196 PMC11000503

[8] Lu CY, Zhang F, Golonski N, Lupton C, Jeffrey P, Wagner AK. State Medicaid reimbursement for medications for chronic hepatitis C infection from 2012 through 2015. Value Health. 2018;21(6):692-697. doi:10.1016/j.jval.2017.09.011 29909874

[9] The Center for Health Law and Policy Innovation of Harvard Law School (CHLPI) and the National Viral Hepatitis Roundtable. Hepatitis C: state of Medicaid access (2024). Accessed April 15, 2024. https://stateofhepc.org/

[10] Centers for Medicare & Medicaid Services. National average drug acquisition cost 2023. Updated December 26, 2023. Accessed June 5, 2024. https://data.medicaid.gov/dataset/4a00010a-132b-4e4d-a611-543c9521280f

[11] Thompson WW, Symum H, Sandul A, . Vital signs: hepatitis C treatment among insured adults—United States, 2019-2020. MMWR Morb Mortal Wkly Rep. 2022;71(32):1011-1017. doi:10.15585/mmwr.mm7132e1 35951484 PMC9400534

[12] Kapadia SN, Jeng PJ, Schackman BR, Bao Y. State Medicaid hepatitis C treatment eligibility criteria and use of direct-acting antivirals. Clin Infect Dis. 2018;66(10):1618-1620. doi:10.1093/cid/cix1062 29206910 PMC6248367

[13] Behrends CN, Gutkind S, Deming R, Fluegge KR, Bresnahan MP, Schackman BR. Impact of removing Medicaid fee-for-service hepatitis C virus (HCV) treatment restrictions on HCV provider experience with Medicaid managed care organizations in New York City. J Urban Health. 2021;98(4):563-569. doi:10.1007/s11524-020-00422-0 32016914 PMC8382819

[14] Herink MC, Geddes J, Vo K, Zaman A, Hartung DM. Effect of relaxing hepatitis C treatment restrictions on direct-acting antiviral use in a Medicaid program: an interrupted time series analysis. J Manag Care Spec Pharm. 2021;27(7):856-864. doi:10.18553/jmcp.2021.27.7.856 34185560 PMC10391280

[15] Davey S, Costello K, Russo M, . Changes in use of hepatitis C direct-acting antivirals after access restrictions were eased by state Medicaid programs. JAMA Health Forum. 2024;5(4):e240302. doi:10.1001/jamahealthforum.2024.0302 38578628 PMC10998155

[16] Centers for Medicare & Medicaid Services. Monthly Medicaid & CHIP application, eligibility determination, and enrollment reports & data. February 2024. Accessed June 5, 2024. https://www.medicaid.gov/medicaid/national-medicaid-chip-program-information/medicaid-chip-enrollment-data/monthly-medicaid-chip-application-eligibility-determination-and-enrollment-reports-data/index.html

[17] Kaiser Family Foundation. Status of state Medicaid expansion decisions. May 8, 2024. Accessed June 5, 2024. https://www.kff.org/medicaid/issue-brief/status-of-state-medicaid-expansion-decisions-interactive-map/

[18] Barua S, Greenwald R, Grebely J, Dore GJ, Swan T, Taylor LE. Restrictions for Medicaid reimbursement of sofosbuvir for the treatment of hepatitis C virus infection in the United States. Ann Intern Med. 2015;163(3):215-223. doi:10.7326/M15-0406 26120969

[19] National Viral Hepatitis Roundtable and Center for Health Law and Policy Innovation. Hepatitis C: the state of Medicaid access preliminary findings: national summary report. November 14, 2016. Accessed October 27, 2023. https://chlpi.org/wp-content/uploads/2013/12/HCV-Report-Card-National-Summary_FINAL.pdf

[20] Ogawa E, Chien N, Kam L, . Association of direct-acting antiviral therapy with liver and nonliver complications and long-term mortality in patients with chronic hepatitis C. JAMA Intern Med. 2023;183(2):97-105. doi:10.1001/jamainternmed.2022.5699 36508196 PMC9856614

[21] Hamill V, Wong S, Benselin J, . Mortality rates among patients successfully treated for hepatitis C in the era of interferon-free antivirals: population based cohort study. BMJ. 2023;382:e074001. doi:10.1136/bmj-2022-074001 37532284 PMC10394680

[22] Zelenev A, Li J, Mazhnaya A, Basu S, Altice FL. Hepatitis C virus treatment as prevention in an extended network of people who inject drugs in the USA: a modelling study. Lancet Infect Dis. 2018;18(2):215-224. doi:10.1016/S1473-3099(17)30676-X 29153265 PMC5860640

[23] Ayoub HH, Abu-Raddad LJ. Impact of treatment on hepatitis C virus transmission and incidence in Egypt: a case for treatment as prevention. J Viral Hepat. 2017;24(6):486-495. doi:10.1111/jvh.12671 28039923

[24] van Santen DK, Sacks-Davis R, Stewart A, ; InCHEHC study group. Treatment as prevention effect of direct-acting antivirals on primary hepatitis C virus incidence: findings from a multinational cohort between 2010 and 2019. EClinicalMedicine. 2022;56:101810. doi:10.1016/j.eclinm.2022.101810 36618902 PMC9816910

[25] Kaplan DE, Serper M, Kaushik A, . Cost-effectiveness of direct-acting antivirals for chronic hepatitis C virus in the United States from a payer perspective. J Manag Care Spec Pharm. 2022;28(10):1138-1148. doi:10.18553/jmcp.2022.28.10.1138 36125059 PMC10373042

[26] Nyberg LM, Kaushik A, Smith N, . Real-world value of direct-acting antivirals for hepatitis C at Kaiser Permanente Southern California. Am J Manag Care. 2023;29(10):e299-e306. doi:10.37765/ajmc.2023.89444 37870551

[27] Chhatwal J, Aaron A, Zhong H, . Projected Health Benefits and Health Care Savings from the United States National Hepatitis C Elimination Initiative. National Bureau of Economic Research Working Paper Series. No. 31139. April 2023.

[28] Heo M, Pericot-Valverde I, Rennert L, . Hepatitis C virus DAA treatment adherence patterns and SVR among people who inject drugs treated in opioid agonist therapy programs. Clin Infect Dis. 2021;73(11):2093-2100. doi:10.1093/cid/ciab334 33876230 PMC8664449

[29] Frankova S, Jandova Z, Jinochova G, Kreidlova M, Merta D, Sperl J. Therapy of chronic hepatitis C in people who inject drugs: focus on adherence. Harm Reduct J. 2021;18(1):69. doi:10.1186/s12954-021-00519-y 34193156 PMC8247095

[30] American Association for the Study of Liver Diseases and the Infectious Disease Society of America. HCV guidance: recommendations for testing, managing, and treating hepatitis C. Accessed November 10, 2021. https://www.hcvguidelines.org/

[31] Fleurence RL, Collins FS. A national hepatitis C elimination program in the United States: a historic opportunity. JAMA. 2023;329(15):1251-1252. doi:10.1001/jama.2023.3692 36892976

