# Supplementary material for: Medicaid Expansion and Restriction Policies for Hepatitis C Treatment
Source: JAMA Netw Open. 2024 Jul 16;7(7):e2422406. doi: 10.1001/jamanetworkopen.2024.22406 (PMC11252896; doi:10.1001/jamanetworkopen.2024.22406)
Supplement: Supplement 1. — eTable 1. Direct-Acting Antiviral National Drug Codes Used for Medicaid Analysis eTable 2. Medicaid Expansion and DAA Restrictive Policy Categorization—US, 2014-2021 eTable 3. Baseline Characteristics of Individuals With Medicaid Filling HCV DAA Prescriptions—US, 2014-2021 eTable 4. Sensitivity Analysis of Multilevel Models of Jurisdictional Medicaid Expansion and DAA Restrictive Policies on DAA Prescription Fill Rates Among Medicaid Recipients Varying Sobriety and Prescriber Restrictions—US, 2014-2021 eFigure 1. Unadjusted Rate of DAA Prescriptions Fills per 100 000 Medicaid Recipients per Year by Jurisdictional Medicaid Expansion Status—US, 2014-2021 eFigure 2. Unadjusted Rate of DAA Prescriptions Fills per 100 000 Medicaid Recipients per Year by Jurisdictional Fibrosis Restrictions—US, 2014-2021 eFigure 3. Unadjusted Rate of DAA Prescriptions Fills per 100 000 Medicaid Recipients per Year by Jurisdictional Sobriety Restrictions—US, 2014-2021 eFigure 4. Unadjusted Rate of DAA Prescriptions Fills per 100 000 Medicaid Recipients per Year by Jurisdictional Prescriber Restrictions—United States, 2014-2021 [file jamanetwopen-e2422406-s001.pdf]

Supplementary Online Content

Furukawa NW, Ingber SZ, Symum H, et al. Medicaid expansion and restriction policies for hepatitis C treatment. *JAMA Netw Open*. 2024;7(7):e2422406. doi:10.1001/jamanetworkopen.2024.22406

eTable 1. Direct-Acting Antiviral National Drug Codes Used for Medicaid Analysis

eTable 2. Medicaid Expansion and DAA Restrictive Policy Categorization—US, 2014-2021

eTable 3. Baseline Characteristics of Individuals With Medicaid Filling HCV DAA Prescriptions—US, 2014-2021

eTable 4. Sensitivity Analysis of Multilevel Models of Jurisdictional Medicaid Expansion and DAA Restrictive Policies on DAA Prescription Fill Rates Among Medicaid Recipients Varying Sobriety and Prescriber Restrictions—US, 2014-2021

eFigure 1. Unadjusted Rate of DAA Prescriptions Fills per 100 000 Medicaid Recipients per Year by Jurisdictional Medicaid Expansion Status—US, 2014-2021

eFigure 2. Unadjusted Rate of DAA Prescriptions Fills per 100 000 Medicaid Recipients per Year by Jurisdictional Fibrosis Restrictions—US, 2014-2021

eFigure 3. Unadjusted Rate of DAA Prescriptions Fills per 100 000 Medicaid Recipients per Year by Jurisdictional Sobriety Restrictions—US, 2014-2021

eFigure 4. Unadjusted Rate of DAA Prescriptions Fills per 100 000 Medicaid Recipients per Year by Jurisdictional Prescriber Restrictions—United States, 2014-2021

This supplementary material has been provided by the authors to give readers additional information about their work.

© 2024 Furukawa NW et al. *JAMA Network Open*.

eTable 1. Direct-Acting Antiviral National Drug Codes Used for Medicaid Analysis

Drug name	Active Ingredients	11-Digit NDC Codes
Sovaldi	Sofosbuvir	61958150101
Sovaldi	Sofosbuvir	61958150201
Sovaldi	Sofosbuvir	61958150202
Sovaldi	Sofosbuvir	61958150301
Sovaldi	Sofosbuvir	61958150401
Sovaldi	Sofosbuvir	61958150501
Harvoni	Ledipasvir/sofosbuvir	61958180501
Harvoni	Ledipasvir/sofosbuvir	61958180401
Harvoni	Ledipasvir/sofosbuvir	61958180301
Harvoni	Ledipasvir/sofosbuvir	61958180202
Harvoni	Ledipasvir/sofosbuvir	61958180101
Viekira pak	Ombitasvir/paritaprevir/ritonavir/dasabuvir	00074309328
Viekira pak	Ombitasvir/paritaprevir/ritonavir/dasabuvir	00074309301
Technivie	Ombitasvir/paritaprevir/ritonavir	00074308228
Zepatier	Elbasvir/grazoprevir	00006307402
Zepatier	Elbasvir/grazoprevir	00006307401
Epclusa	Sofosbuvir/velpatasvir	61958220101
Epclusa	Sofosbuvir/velpatasvir	61958220201
Epclusa	Sofosbuvir/velpatasvir	61958220202
Epclusa	Sofosbuvir/velpatasvir	61958220203
Epclusa	Sofosbuvir/velpatasvir	61958220301
Epclusa	Sofosbuvir/velpatasvir	61958220401
Epclusa	Sofosbuvir/velpatasvir	61958220402
Epclusa	Sofosbuvir/velpatasvir	61958220501
Viekira XR	Dasabuvir/ombitasvir/paritaprevir/ritonavir	00074006328
Viekira XR	Dasabuvir/ombitasvir/paritaprevir/ritonavir	00074006301
Vosevi	Sofosbuvir/velpatasvir/voxilaprevir	61958240101
Mavyret	Glecaprevir/pibrentasvir	00074262528
Mavyret	Glecaprevir/pibrentasvir	00074262501
Mavyret	Glecaprevir/pibrentasvir	00074262556
Mavyret	Glecaprevir/pibrentasvir	00074262580
Mavyret	Glecaprevir/pibrentasvir	00074260028
Mavyret	Glecaprevir/pibrentasvir	00074262584
Mavyret	Glecaprevir/pibrentasvir	00074308228
Epclusa authorized generic	Velpatasvir/sofosbuvir	72626270101
Harvoni authorized generic	Ledipasvir/sofosbuvir	72626260101
Incivek	Telaprevir	51167010001
Incivek	Telaprevir	51167010003
Olysio	Simeprevir sodium	59676022528
Victrelis	Boceprevir	00085031402
Daklinza	Daclatasvir dihydrochloride	0003001101
Daklinza	Daclatasvir dihydrochloride	0003021301
Daklinza	Daclatasvir dihydrochloride	0003021501

© 2024 Furukawa NW et al. *JAMA Network Open*.

eTable 2. Medicaid Expansion and DAA Restrictive Policy Categorization—US, 2014-2021

State	Year	Medicaid	Fibrosis Restrictions	Sobriety Restrictions	Provider Restrictions	Prior Authorization
AL	2014	Not Expanded	Unknown	6–12 Months	Unknown	Required
AL	2015	Not Expanded	Unknown	6–12 Months	Unknown	Required
AL	2016	Not Expanded	Unknown	6–12 Months	Unknown	Required
AL	2017	Not Expanded	F1–F2	6–12 Months	None	Required
AL	2018	Not Expanded	F1–F2	6–12 Months	None	Required
AL	2019	Not Expanded	F1–F2	6–12 Months	None	Required
AL	2020	Not Expanded	F1–F2	6–12 Months	None	Required
AL	2021	Not Expanded	None	6–12 Months	None	Required
AK	2014	Not Expanded	F3–F4	1–5 Months	Unknown	Required
AK	2015	Expanded	F3–F4	1–5 Months	Unknown	Required
AK	2016	Expanded	F1–F2	1–5 Months	Specialist Consult	Required
AK	2017	Expanded	None	Screening & Counseling	None	Required
AK	2018	Expanded	None	Screening & Counseling	None	Required
AK	2019	Expanded	None	Screening & Counseling	None	Required
AK	2020	Expanded	None	Screening & Counseling	None	Required
AK	2021	Expanded	None	Screening & Counseling	None	Required
AZ	2014	Expanded	F3–F4	6–12 Months	Specialist Consult	Required
AZ	2015	Expanded	F3–F4	6–12 Months	Specialist Consult	Required
AZ	2016	Expanded	None	6–12 Months	Specialist Consult	Required
AZ	2017	Expanded	F1–F2	1–5 Months	Specialist Consult	Required
AZ	2018	Expanded	None	1–5 Months	Specialist Consult	Required
AZ	2019	Expanded	None	1–5 Months	Specialist Consult	Required
AZ	2020	Expanded	None	1–5 Months	Specialist Consult	Required
AZ	2021	Expanded	None	1–5 Months	Specialist Consult	Required
AR	2014	Expanded	F3–F4	Screening & Counseling	Unknown	Required
AR	2015	Expanded	F3–F4	Screening & Counseling	Unknown	Required
AR	2016	Expanded	F3–F4	6–12 Months	Unknown	Required
AR	2017	Expanded	F3–F4	6–12 Months	Specialist Required	Required
AR	2018	Expanded	F3–F4	6–12 Months	Specialist Required	Required
AR	2019	Expanded	F3–F4	6–12 Months	Specialist Required	Required
AR	2020	Expanded	F3–F4	6–12 Months	Specialist Required	Required
AR	2021	Expanded	F3–F4	6–12 Months	Specialist Required	Required
CA	2014	Expanded	F3–F4	6–12 Months	Specialist Consult	Required
CA	2015	Expanded	F3–F4	6–12 Months	Specialist Consult	Required
CA	2016	Expanded	F1–F2	Unknown	Unknown	Required
CA	2017	Expanded	F1–F2	None	None	Required
CA	2018	Expanded	None	None	None	Required
CA	2019	Expanded	None	None	None	Required
CA	2020	Expanded	None	None	None	Required
CA	2021	Expanded	None	None	None	Removed

© 2024 Furukawa NW et al. *JAMA Network Open*.

State	Year	Medicaid	Fibrosis Restrictions	Sobriety Restrictions	Provider Restrictions	Prior Authorization
CO	2014	Expanded	F3–F4	6–12 Months	Specialist Consult	Required
CO	2015	Expanded	F3–F4	6–12 Months	Specialist Consult	Required
CO	2016	Expanded	F3–F4	6–12 Months	Specialist Consult	Required
CO	2017	Expanded	F1–F2	Screening & Counseling	Specialist Consult	Required
CO	2018	Expanded	None	Screening & Counseling	Specialist Consult	Required
CO	2019	Expanded	None	Screening & Counseling	Specialist Consult	Required
CO	2020	Expanded	None	Screening & Counseling	Specialist Consult	Required
CO	2021	Expanded	None	Screening & Counseling	Specialist Consult	Required
CT	2014	Expanded	F3–F4	Screening & Counseling	Specialist Consult	Required
CT	2015	Expanded	None	None	None	Required
CT	2016	Expanded	None	None	None	Required
CT	2017	Expanded	None	None	None	Required
CT	2018	Expanded	None	None	None	Required
CT	2019	Expanded	None	None	None	Required
CT	2020	Expanded	None	None	None	Required
CT	2021	Expanded	None	None	None	Required
DE	2014	Expanded	F3–F4	1–5 Months	Unknown	Required
DE	2015	Expanded	F3–F4	1–5 Months	Unknown	Required
DE	2016	Expanded	F3–F4	Screening & Counseling	Unknown	Required
DE	2017	Expanded	F1–F2	Screening & Counseling	None	Required
DE	2018	Expanded	None	Screening & Counseling	None	Required
DE	2019	Expanded	None	Screening & Counseling	None	Required
DE	2020	Expanded	None	Screening & Counseling	None	Required
DE	2021	Expanded	None	Screening & Counseling	None	Required
DC	2014	Expanded	F3–F4	1–5 Months	Specialist Consult	Required
DC	2015	Expanded	F1–F2	None	Specialist Consult	Required
DC	2016	Expanded	F1–F2	None	Specialist Consult	Required
DC	2017	Expanded	None	Screening & Counseling	Specialist Consult	Required
DC	2018	Expanded	None	Screening & Counseling	Specialist Consult	Required
DC	2019	Expanded	None	Screening & Counseling	Specialist Consult	Required
DC	2020	Expanded	None	Screening & Counseling	Specialist Consult	Required
DC	2021	Expanded	None	Screening & Counseling	Specialist Consult	Required
FL	2014	Not Expanded	F3–F4	1–5 Months	Specialist Required	Required
FL	2015	Not Expanded	F3–F4	1–5 Months	Specialist Required	Required
FL	2016	Not Expanded	None	1–5 Months	Specialist Consult	Required
FL	2017	Not Expanded	None	1–5 Months	Specialist Consult	Required
FL	2018	Not Expanded	None	1–5 Months	Specialist Consult	Required
FL	2019	Not Expanded	None	1–5 Months	Specialist Consult	Required
FL	2020	Not Expanded	None	1–5 Months	None	Required
FL	2021	Not Expanded	None	1–5 Months	None	Required
GA	2014	Not Expanded	Unknown	Unknown	Unknown	Required
GA	2015	Not Expanded	Unknown	Unknown	Unknown	Required

© 2024 Furukawa NW et al. *JAMA Network Open*.

State	Year	Medicaid	Fibrosis Restrictions	Sobriety Restrictions	Provider Restrictions	Prior Authorization
GA	2016	Not Expanded	None	Screening & Counseling	Unknown	Required
GA	2017	Not Expanded	None	Screening & Counseling	None	Required
GA	2018	Not Expanded	None	Screening & Counseling	None	Required
GA	2019	Not Expanded	None	Screening & Counseling	None	Required
GA	2020	Not Expanded	None	Screening & Counseling	None	Required
GA	2021	Not Expanded	None	Screening & Counseling	None	Required
HI	2014	Expanded	Unknown	Unknown	Unknown	Required
HI	2015	Expanded	Unknown	Unknown	Unknown	Required
HI	2016	Expanded	F3–F4	1–5 Months	Specialist Consult	Required
HI	2017	Expanded	F1–F2	1–5 Months	Specialist Consult	Required
HI	2018	Expanded	None	None	None	Required
HI	2019	Expanded	None	None	Specialist Consult	Required
HI	2020	Expanded	None	Screening & Counseling	Specialist Consult	Required
HI	2021	Expanded	None	Screening & Counseling	Specialist Consult	Required
ID	2014	Not Expanded	F3–F4	6–12 Months	Specialist Consult	Required
ID	2015	Not Expanded	F3–F4	6–12 Months	Specialist Consult	Required
ID	2016	Not Expanded	F1–F2	6–12 Months	Specialist Consult	Required
ID	2017	Not Expanded	F1–F2	6–12 Months	Specialist Consult	Required
ID	2018	Not Expanded	None	6–12 Months	Specialist Consult	Required
ID	2019	Not Expanded	None	Screening & Counseling	None	Required
ID	2020	Expanded	None	Screening & Counseling	None	Required
ID	2021	Expanded	None	Screening & Counseling	None	Required
IL	2014	Expanded	F3–F4	6–12 Months	Specialist Consult	Required
IL	2015	Expanded	F3–F4	6–12 Months	Specialist Consult	Required
IL	2016	Expanded	None	6–12 Months	Specialist Consult	Required
IL	2017	Expanded	F3–F4	Screening & Counseling	Specialist Consult	Required
IL	2018	Expanded	None	Screening & Counseling	Specialist Consult	Required
IL	2019	Expanded	None	Screening & Counseling	Specialist Consult	Required
IL	2020	Expanded	None	Screening & Counseling	Specialist Consult	Required
IL	2021	Expanded	None	Screening & Counseling	Specialist Consult	Required
IN	2014	Not Expanded	F3–F4	Screening & Counseling	Specialist Required	Required
IN	2015	Expanded	F3–F4	Screening & Counseling	Specialist Consult	Required
IN	2016	Expanded	F3–F4	Unknown	Specialist Consult	Required
IN	2017	Expanded	F1–F2	None	Specialist Consult	Required
IN	2018	Expanded	F1–F2	None	Specialist Consult	Required
IN	2019	Expanded	None	None	Specialist Consult	Required
IN	2020	Expanded	None	None	Specialist Consult	Removed
IN	2021	Expanded	None	None	None	Removed
IA	2014	Expanded	F3–F4	1–5 Months	Specialist Required	Required
IA	2015	Expanded	F3–F4	1–5 Months	Specialist Required	Required
IA	2016	Expanded	F3–F4	1–5 Months	Specialist Required	Required
IA	2017	Expanded	F3–F4	1–5 Months	Specialist Required	Required

© 2024 Furukawa NW et al. *JAMA Network Open*.

State	Year	Medicaid	Fibrosis Restrictions	Sobriety Restrictions	Provider Restrictions	Prior Authorization
IA	2018	Expanded	F3–F4	1–5 Months	Specialist Required	Required
IA	2019	Expanded	F1–F2	1–5 Months	Specialist Consult	Required
IA	2020	Expanded	None	1–5 Months	Specialist Consult	Required
IA	2021	Expanded	None	1–5 Months	Specialist Consult	Required
KS	2014	Not Expanded	Unknown	Unknown	Unknown	Required
KS	2015	Not Expanded	Unknown	Unknown	Unknown	Required
KS	2016	Not Expanded	F3–F4	6–12 Months	Specialist Consult	Required
KS	2017	Not Expanded	F3–F4	6–12 Months	Specialist Consult	Required
KS	2018	Not Expanded	None	1–5 Months	Specialist Consult	Required
KS	2019	Not Expanded	None	1–5 Months	None	Required
KS	2020	Not Expanded	None	1–5 Months	None	Required
KS	2021	Not Expanded	None	None	None	Required
KY	2014	Expanded	F3–F4	6–12 Months	Specialist Consult	Required
KY	2015	Expanded	F3–F4	6–12 Months	Specialist Consult	Required
KY	2016	Expanded	F3–F4	Unknown	Unknown	Required
KY	2017	Expanded	F1–F2	6–12 Months	Specialist Consult	Required
KY	2018	Expanded	None	Screening & Counseling	Specialist Consult	Required
KY	2019	Expanded	None	Screening & Counseling	Specialist Consult	Required
KY	2020	Expanded	None	Screening & Counseling	None	Required
KY	2021	Expanded	None	None	None	Required
LA	2014	Not Expanded	F3–F4	6–12 Months	Specialist Required	Required
LA	2015	Not Expanded	F3–F4	6–12 Months	Specialist Required	Required
LA	2016	Expanded	F3–F4	6–12 Months	Specialist Required	Required
LA	2017	Expanded	F3–F4	6–12 Months	Specialist Required	Required
LA	2018	Expanded	F3–F4	Screening & Counseling	None	Required
LA	2019	Expanded	None	None	None	Removed
LA	2020	Expanded	None	None	None	Removed
LA	2021	Expanded	None	None	None	Removed
ME	2014	Not Expanded	F1–F2	Screening & Counseling	Specialist Required	Required
ME	2015	Not Expanded	F1–F2	Screening & Counseling	Unknown	Required
ME	2016	Not Expanded	Unknown	6–12 Months	Specialist Consult	Required
ME	2017	Not Expanded	None	6–12 Months	Specialist Consult	Required
ME	2018	Not Expanded	None	None	Specialist Consult	Required
ME	2019	Expanded	None	None	Specialist Consult	Required
ME	2020	Expanded	None	None	None	Required
ME	2021	Expanded	None	None	None	Required
MD	2014	Expanded	F1–F2	6–12 Months	Specialist Required	Required
MD	2015	Expanded	F1–F2	6–12 Months	Specialist Required	Required
MD	2016	Expanded	F1–F2	6–12 Months	Specialist Required	Required
MD	2017	Expanded	F1–F2	Screening & Counseling	Specialist Consult	Required
MD	2018	Expanded	F1–F2	Screening & Counseling	Specialist Consult	Required
MD	2019	Expanded	F1–F2	Screening & Counseling	Specialist Consult	Required

© 2024 Furukawa NW et al. *JAMA Network Open*.

State	Year	Medicaid	Fibrosis Restrictions	Sobriety Restrictions	Provider Restrictions	Prior Authorization
MD	2020	Expanded	None	Screening & Counseling	None	Required
MD	2021	Expanded	None	Screening & Counseling	None	Required
MA	2014	Expanded	Unknown	6–12 Months	Unknown	Required
MA	2015	Expanded	F3–F4	6–12 Months	Specialist Required	Required
MA	2016	Expanded	None	None	None	Required
MA	2017	Expanded	None	None	None	Required
MA	2018	Expanded	None	None	None	Required
MA	2019	Expanded	None	None	None	Required
MA	2020	Expanded	None	None	None	Required
MA	2021	Expanded	None	None	None	Required
MI	2014	Expanded	Unknown	Unknown	Unknown	Required
MI	2015	Expanded	Unknown	Unknown	Unknown	Required
MI	2016	Expanded	F3–F4	Unknown	Specialist Consult	Required
MI	2017	Expanded	F1–F2	6–12 Months	Specialist Consult	Required
MI	2018	Expanded	F1–F2	Screening & Counseling	Specialist Consult	Required
MI	2019	Expanded	None	Screening & Counseling	Specialist Consult	Required
MI	2020	Expanded	None	Screening & Counseling	Specialist Consult	Required
MI	2021	Expanded	None	None	None	Removed
MN	2014	Expanded	Unknown	None	Unknown	Required
MN	2015	Expanded	Unknown	None	Unknown	Required
MN	2016	Expanded	F1–F2	6–12 Months	Specialist Consult	Required
MN	2017	Expanded	None	6–12 Months	Specialist Consult	Required
MN	2018	Expanded	None	6–12 Months	Specialist Consult	Required
MN	2019	Expanded	None	6–12 Months	Specialist Consult	Required
MN	2020	Expanded	None	Screening & Counseling	None	Required
MN	2021	Expanded	None	Screening & Counseling	None	Required
MS	2014	Not Expanded	Unknown	6–12 Months	Specialist Consult	Required
MS	2015	Not Expanded	Unknown	6–12 Months	Specialist Consult	Required
MS	2016	Not Expanded	Unknown	6–12 Months	Specialist Consult	Required
MS	2017	Not Expanded	None	6–12 Months	Specialist Consult	Required
MS	2018	Not Expanded	None	6–12 Months	Specialist Consult	Required
MS	2019	Not Expanded	None	6–12 Months	Specialist Consult	Required
MS	2020	Not Expanded	None	6–12 Months	Specialist Consult	Required
MS	2021	Not Expanded	None	6–12 Months	Specialist Consult	Required
MO	2014	Not Expanded	F3–F4	1–5 Months	Unknown	Required
MO	2015	Not Expanded	F3–F4	1–5 Months	Unknown	Required
MO	2016	Not Expanded	F1–F2	1–5 Months	Unknown	Required
MO	2017	Not Expanded	None	None	None	Required
MO	2018	Not Expanded	None	None	None	Required
MO	2019	Not Expanded	None	None	None	Required
MO	2020	Not Expanded	None	None	None	Required
MO	2021	Expanded	None	None	None	Missouri

© 2024 Furukawa NW et al. *JAMA Network Open*.

State	Year	Medicaid	Fibrosis Restrictions	Sobriety Restrictions	Provider Restrictions	Prior Authorization
MT	2014	Not Expanded	F3–F4	6–12 Months	Specialist Consult	Required
MT	2015	Not Expanded	F3–F4	6–12 Months	Specialist Consult	Required
MT	2016	Expanded	F3–F4	6–12 Months	Specialist Consult	Required
MT	2017	Expanded	F3–F4	6–12 Months	Specialist Required	Required
MT	2018	Expanded	F3–F4	6–12 Months	Specialist Required	Required
MT	2019	Expanded	F3–F4	6–12 Months	Specialist Required	Required
MT	2020	Expanded	None	Screening & Counseling	None	Required
MT	2021	Expanded	None	Screening & Counseling	None	Required
NE	2014	Not Expanded	F3–F4	6–12 Months	Unknown	Required
NE	2015	Not Expanded	F3–F4	6–12 Months	Unknown	Required
NE	2016	Not Expanded	F3–F4	1–5 Months	Unknown	Required
NE	2017	Not Expanded	F3–F4	6–12 Months	None	Required
NE	2018	Not Expanded	F3–F4	6–12 Months	None	Required
NE	2019	Not Expanded	F1–F2	6–12 Months	None	Required
NE	2020	Expanded	F1–F2	6–12 Months	None	Required
NE	2021	Expanded	None	6–12 Months	None	Required
NV	2014	Expanded	None	None	None	Required
NV	2015	Expanded	None	None	None	Required
NV	2016	Expanded	None	Unknown	None	Required
NV	2017	Expanded	None	None	None	Required
NV	2018	Expanded	None	None	None	Required
NV	2019	Expanded	None	None	Specialist Consult	Required
NV	2020	Expanded	None	None	Specialist Consult	Required
NV	2021	Expanded	None	None	Specialist Consult	Required
NH	2014	Expanded	F3–F4	Screening & Counseling	Specialist Required	Required
NH	2015	Expanded	F3–F4	6–12 Months	Specialist Required	Required
NH	2016	Expanded	None	Screening & Counseling	Specialist Consult	Required
NH	2017	Expanded	None	Screening & Counseling	Specialist Consult	Required
NH	2018	Expanded	None	Screening & Counseling	Specialist Consult	Required
NH	2019	Expanded	None	Screening & Counseling	Specialist Consult	Required
NH	2020	Expanded	None	Screening & Counseling	Specialist Consult	Required
NH	2021	Expanded	None	Screening & Counseling	Specialist Consult	Required
NJ	2014	Expanded	Unknown	Unknown	Unknown	Required
NJ	2015	Expanded	F3–F4	1–5 Months	Unknown	Required
NJ	2016	Expanded	F3–F4	1–5 Months	Unknown	Required
NJ	2017	Expanded	F1–F2	None	Unknown	Required
NJ	2018	Expanded	None	None	None	Required
NJ	2019	Expanded	None	None	Specialist Required	Required
NJ	2020	Expanded	None	None	Specialist Required	Required
NJ	2021	Expanded	None	None	None	Required
NM	2014	Expanded	Unknown	Unknown	Unknown	Required
NM	2015	Expanded	Unknown	Unknown	Unknown	Required

© 2024 Furukawa NW et al. *JAMA Network Open*.

State	Year	Medicaid	Fibrosis Restrictions	Sobriety Restrictions	Provider Restrictions	Prior Authorization
NM	2016	Expanded	F1–F2	Screening & Counseling	Unknown	Required
NM	2017	Expanded	F1–F2	Screening & Counseling	None	Required
NM	2018	Expanded	None	Screening & Counseling	None	Required
NM	2019	Expanded	None	Screening & Counseling	None	Required
NM	2020	Expanded	None	Screening & Counseling	None	Required
NM	2021	Expanded	None	None	None	Required
NY	2014	Expanded	F3–F4	Screening & Counseling	Specialist Required	Required
NY	2015	Expanded	Unknown	Screening & Counseling	Unknown	Required
NY	2016	Expanded	None	Screening & Counseling	Specialist Consult	Required
NY	2017	Expanded	None	Screening & Counseling	Specialist Consult	Required
NY	2018	Expanded	None	Screening & Counseling	None	Required
NY	2019	Expanded	None	Screening & Counseling	None	Required
NY	2020	Expanded	None	None	None	Removed
NY	2021	Expanded	None	None	None	Removed
NC	2014	Not Expanded	Unknown	Screening & Counseling	Unknown	Required
NC	2015	Not Expanded	Unknown	Screening & Counseling	Unknown	Required
NC	2016	Not Expanded	F1–F2	Screening & Counseling	Unknown	Required
NC	2017	Not Expanded	None	Screening & Counseling	None	Required
NC	2018	Not Expanded	None	Screening & Counseling	None	Required
NC	2019	Not Expanded	None	Screening & Counseling	None	Required
NC	2020	Not Expanded	None	Screening & Counseling	None	Required
NC	2021	Not Expanded	None	None	None	Required
ND	2014	Expanded	Unknown	Unknown	Unknown	Required
ND	2015	Expanded	Unknown	Unknown	Unknown	Required
ND	2016	Expanded	F1–F2	6–12 Months	Specialist Consult	Required
ND	2017	Expanded	None	6–12 Months	Specialist Consult	Required
ND	2018	Expanded	None	1–5 Months	Specialist Consult	Required
ND	2019	Expanded	None	1–5 Months	Specialist Consult	Required
ND	2020	Expanded	None	1–5 Months	Specialist Consult	Required
ND	2021	Expanded	None	1–5 Months	Specialist Consult	Required
OH	2014	Expanded	F3–F4	Unknown	Specialist Required	Required
OH	2015	Expanded	F3–F4	6–12 Months	Specialist Required	Required
OH	2016	Expanded	F3–F4	6–12 Months	Specialist Required	Required
OH	2017	Expanded	F1–F2	6–12 Months	Specialist Required	Required
OH	2018	Expanded	F1–F2	6–12 Months	Specialist Required	Required
OH	2019	Expanded	None	6–12 Months	Specialist Consult	Required
OH	2020	Expanded	None	None	Specialist Consult	Required
OH	2021	Expanded	None	None	Specialist Consult	Required
OK	2014	Not Expanded	F1–F2	6–12 Months	Specialist Consult	Required
OK	2015	Not Expanded	F1–F2	6–12 Months	Specialist Consult	Required
OK	2016	Not Expanded	F1–F2	6–12 Months	Specialist Consult	Required
OK	2017	Not Expanded	F1–F2	6–12 Months	Specialist Consult	Required

© 2024 Furukawa NW et al. *JAMA Network Open*.

State	Year	Medicaid	Fibrosis Restrictions	Sobriety Restrictions	Provider Restrictions	Prior Authorization
OK	2018	Not Expanded	None	Screening & Counseling	Specialist Consult	Required
OK	2019	Not Expanded	None	Screening & Counseling	Specialist Consult	Required
OK	2020	Not Expanded	None	Screening & Counseling	Specialist Consult	Required
OK	2021	Expanded	None	Screening & Counseling	Specialist Consult	Required
OR	2014	Expanded	F3–F4	6–12 Months	Specialist Consult	Required
OR	2015	Expanded	F3–F4	6–12 Months	Specialist Consult	Required
OR	2016	Expanded	F3–F4	Screening & Counseling	Specialist Consult	Required
OR	2017	Expanded	F3–F4	6–12 Months	Specialist Consult	Required
OR	2018	Expanded	F1–F2	6–12 Months	Specialist Consult	Required
OR	2019	Expanded	None	None	None	Required
OR	2020	Expanded	None	None	None	Required
OR	2021	Expanded	None	None	None	Required
PA	2014	Not Expanded	F3–F4	6–12 Months	Specialist Required	Required
PA	2015	Expanded	F3–F4	Unknown	Specialist Required	Required
PA	2016	Expanded	F1–F2	Screening & Counseling	Specialist Required	Required
PA	2017	Expanded	F1–F2	Screening & Counseling	Specialist Required	Required
PA	2018	Expanded	None	Screening & Counseling	Specialist Consult	Required
PA	2019	Expanded	None	Screening & Counseling	None	Required
PA	2020	Expanded	None	Screening & Counseling	None	Required
PA	2021	Expanded	None	Screening & Counseling	None	Required
RI	2014	Expanded	F3–F4	6–12 Months	Specialist Required	Required
RI	2015	Expanded	F3–F4	6–12 Months	Specialist Required	Required
RI	2016	Expanded	F3–F4	6–12 Months	Specialist Required	Required
RI	2017	Expanded	F3–F4	Screening & Counseling	Specialist Required	Required
RI	2018	Expanded	None	None	None	Required
RI	2019	Expanded	None	None	None	Required
RI	2020	Expanded	None	None	None	Required
RI	2021	Expanded	None	None	None	Removed
SC	2014	Not Expanded	Unknown	Unknown	Unknown	Required
SC	2015	Not Expanded	Unknown	Unknown	Unknown	Required
SC	2016	Not Expanded	F3–F4	Screening & Counseling	Unknown	Required
SC	2017	Not Expanded	None	Screening & Counseling	Specialist Consult	Required
SC	2018	Not Expanded	None	Screening & Counseling	Specialist Consult	Required
SC	2019	Not Expanded	None	Screening & Counseling	Specialist Consult	Required
SC	2020	Not Expanded	None	Screening & Counseling	Specialist Consult	Required
SC	2021	Not Expanded	None	Screening & Counseling	Specialist Consult	Required
SD	2014	Not Expanded	F3–F4	6–12 Months	Specialist Consult	Required
SD	2015	Not Expanded	F3–F4	6–12 Months	Specialist Consult	Required
SD	2016	Not Expanded	F3–F4	6–12 Months	Specialist Required	Required
SD	2017	Not Expanded	F3–F4	6–12 Months	Specialist Required	Required
SD	2018	Not Expanded	F3–F4	6–12 Months	Specialist Consult	Required
SD	2019	Not Expanded	F3–F4	6–12 Months	Specialist Consult	Required

© 2024 Furukawa NW et al. *JAMA Network Open*.

State	Year	Medicaid	Fibrosis Restrictions	Sobriety Restrictions	Provider Restrictions	Prior Authorization
SD	2020	Not Expanded	F3–F4	6–12 Months	Specialist Consult	Required
SD	2021	Not Expanded	F1–F2	6–12 Months	Specialist Consult	Required
TN	2014	Not Expanded	F3–F4	6–12 Months	Specialist Required	Required
TN	2015	Not Expanded	F3–F4	6–12 Months	Specialist Required	Required
TN	2016	Not Expanded	F1–F2	6–12 Months	Specialist Required	Required
TN	2017	Not Expanded	F1–F2	6–12 Months	Specialist Required	Required
TN	2018	Not Expanded	F1–F2	6–12 Months	Specialist Consult	Required
TN	2019	Not Expanded	None	6–12 Months	None	Required
TN	2020	Not Expanded	None	6–12 Months	None	Required
TN	2021	Not Expanded	None	6–12 Months	None	Required
TX	2014	Not Expanded	Unknown	Unknown	Unknown	Required
TX	2015	Not Expanded	Unknown	Unknown	Unknown	Required
TX	2016	Not Expanded	F3–F4	1–5 Months	Specialist Required	Required
TX	2017	Not Expanded	F3–F4	1–5 Months	Specialist Consult	Required
TX	2018	Not Expanded	F3–F4	1–5 Months	Specialist Consult	Required
TX	2019	Not Expanded	F3–F4	1–5 Months	Specialist Consult	Required
TX	2020	Not Expanded	F3–F4	1–5 Months	Specialist Consult	Required
TX	2021	Not Expanded	None	None	None	Required
UT	2014	Not Expanded	Unknown	Unknown	Unknown	Required
UT	2015	Not Expanded	Unknown	Unknown	Unknown	Required
UT	2016	Not Expanded	F1–F2	Unknown	Specialist Consult	Required
UT	2017	Not Expanded	F1–F2	None	Specialist Consult	Required
UT	2018	Not Expanded	None	None	Specialist Consult	Required
UT	2019	Not Expanded	None	None	Specialist Consult	Required
UT	2020	Expanded	None	None	Specialist Consult	Required
UT	2021	Expanded	None	None	None	Required
VT	2014	Expanded	F3–F4	Screening & Counseling	Unknown	Required
VT	2015	Expanded	F3–F4	Screening & Counseling	Unknown	Required
VT	2016	Expanded	F3–F4	6–12 Months	Specialist Required	Required
VT	2017	Expanded	None	None	Specialist Consult	Required
VT	2018	Expanded	None	None	Specialist Consult	Required
VT	2019	Expanded	None	None	Specialist Consult	Required
VT	2020	Expanded	None	None	Specialist Consult	Required
VT	2021	Expanded	None	None	None	Required
VA	2014	Not Expanded	F3–F4	Screening & Counseling	Specialist Consult	Required
VA	2015	Not Expanded	F3–F4	Screening & Counseling	Specialist Consult	Required
VA	2016	Not Expanded	F1–F2	Screening & Counseling	Specialist Consult	Required
VA	2017	Not Expanded	None	Screening & Counseling	Specialist Consult	Required
VA	2018	Not Expanded	None	Screening & Counseling	Specialist Consult	Required
VA	2019	Expanded	None	Screening & Counseling	Specialist Consult	Required
VA	2020	Expanded	None	None	None	Required
VA	2021	Expanded	None	None	None	Required

© 2024 Furukawa NW et al. *JAMA Network Open*.

State	Year	Medicaid	Fibrosis Restrictions	Sobriety Restrictions	Provider Restrictions	Prior Authorization
WA	2014	Expanded	F3–F4	1–5 Months	Specialist Required	Required
WA	2015	Expanded	F3–F4	1–5 Months	Unknown	Required
WA	2016	Expanded	None	Unknown	Specialist Consult	Required
WA	2017	Expanded	None	None	Specialist Consult	Required
WA	2018	Expanded	None	None	Specialist Consult	Required
WA	2019	Expanded	None	None	None	Removed
WA	2020	Expanded	None	None	None	Removed
WA	2021	Expanded	None	None	None	Removed
WV	2014	Expanded	F3–F4	6–12 Months	Specialist Consult	Required
WV	2015	Expanded	F3–F4	6–12 Months	Specialist Consult	Required
WV	2016	Expanded	F3–F4	1–5 Months	Specialist Consult	Required
WV	2017	Expanded	F1–F2	1–5 Months	Specialist Consult	Required
WV	2018	Expanded	F1–F2	1–5 Months	Specialist Consult	Required
WV	2019	Expanded	None	1–5 Months	Specialist Consult	Required
WV	2020	Expanded	None	1–5 Months	Specialist Consult	Required
WV	2021	Expanded	None	1–5 Months	Specialist Consult	Required
WI	2014	Not Expanded	F3–F4	6–12 Months	Specialist Required	Required
WI	2015	Not Expanded	F3–F4	6–12 Months	Specialist Consult	Required
WI	2016	Not Expanded	F1–F2	6–12 Months	Specialist Consult	Required
WI	2017	Not Expanded	None	6–12 Months	None	Required
WI	2018	Not Expanded	None	Screening & Counseling	None	Required
WI	2019	Not Expanded	None	None	None	Required
WI	2020	Not Expanded	None	None	None	Removed
WI	2021	Not Expanded	None	None	None	Removed
WY	2014	Not Expanded	None	1–5 Months	Unknown	Required
WY	2015	Not Expanded	None	1–5 Months	Unknown	Required
WY	2016	Not Expanded	None	1–5 Months	Unknown	Required
WY	2017	Not Expanded	None	1–5 Months	None	Required
WY	2018	Not Expanded	None	1–5 Months	None	Required
WY	2019	Not Expanded	None	1–5 Months	None	Required
WY	2020	Not Expanded	None	Screening & Counseling	None	Required
WY	2021	Not Expanded	None	Screening & Counseling	None	Required

Footnote: DAA restriction policies were collected from publications, National Viral Hepatitis Round Table and Harvard Center for Health Law and Policy Innovation State of Hepatitis C reports, and Medicaid documents. The earliest DAA policy restriction change that happened in the calendar year was applied to the jurisdiction-year the policy change happened.

© 2024 Furukawa NW et al. *JAMA Network Open*.

eTable 3. Baseline Characteristics of Individuals With Medicaid Filling HCV DAA Prescriptions—US, 2014-2021

**Characteristics**	**Individuals with Medicaid Prescribed DAAs n (%)**
**Total**	381,373
<45 years	152,178 (39.9)
45-64 years	218,479 (57.3)
≥65 years	7,827 (2.1)
Missing	2,889 (0.8)
**Sex**	
Female	151,224 (39.7)
Male	223,804 (58.7)
Missing	6,345 (1.7)
**Race/Ethnicity**	
White, non-Hispanic	199,159 (52.2)
Black, non-Hispanic	58,023 (15.2)
Hispanic	39,632 (10.4)
Asian	6,148 (1.6)
Others	6,757 (1.8)
Missing	71,654 (18.8)
**Census Region**	
West	106,159 (27.8)
Midwest	61,094 (16.0)
South	94,914 (24.9)
Northeast	119,206 (31.3)

© 2024 Furukawa NW et al. *JAMA Network Open*.

eTable 4. Sensitivity Analysis of Multilevel Models of Jurisdictional Medicaid Expansion and DAA Restrictive Policies on DAA Prescription Fill Rates Among Medicaid Recipients Varying Sobriety and Prescriber Restrictions—US, 2014-2021

	Bivariate model		Model 1[a]		Model 2[b]		Model 3[c]	
	**Relative Risk (95% CI)**	***P* value**	**Relative Risk (95% CI)**	***P* value**	**Relative Risk (95% CI)**	***P* value**	**Relative Risk (95% CI)**	***P* value**
**Jurisdiction with Medicaid Expansion**								
Yes	Ref		Ref		Ref		Ref	
No	0.45 (0.35 to 0.57)	<.001	0.56 (0.52,0.61)	<.001	0.58 (0.54,0.62)	<.001	0.54 (0.5,0.58)	<.001
**Jurisdictions with Fibrosis Restrictions**								
None	Ref		Ref		Ref		Ref	
F1–F2	0.64 (0.49 to 0.84)	.002	0.62 (0.59,0.66)	<.001	0.65 (0.62,0.69)	<.001	0.61 (0.58,0.64)	<.001
F3–F4	0.34 (0.27 to 0.42)	<.001	0.39 (0.37,0.43)	<.001	0.41 (0.38,0.44)	<.001	0.35 (0.32,0.37)	<.001
**Jurisdictions with Sobriety Restrictions**								
None	Ref		Ref		Ref			
Screening/Counseling	0.78 (0.61 to 0.99)	.04	0.87 (0.83,0.92)	<.001	0.97 (0.90,1.06)	0.51		
1–5 months	0.42 (0.28 to 0.63)	<.001	1.08 (0.97,1.2)	.14	0.69 (0.64,0.73)	<.001		
6–12 months	0.34 (0.27 to 0.43)	<.001	0.65 (0.61,0.71	<.001	0.91 (0.87,0.96)	<.001		
**Jurisdictions with Provider Restrictions**								
None	Ref		Ref				Ref	
Specialist Consult	0.68 (0.54 to 0.87)	.002	1.05 (1.00,1.10)	0.05			0.99 (0.95,1.04)	0.74
Specialist Required	0.46 (0.32 to 0.65)	<.001	1.07 (0.99,1.15)	0.08			0.96 (0.9,1.03)	0.28

a. Model 1: Multilevel Poisson regression model with random effects for state and year.

b. Model 2: Multilevel Poisson regression model with random effects for state and year. Provider restrictions were excluded from the model.

c. Model 3: Multilevel Poisson regression model with random effects for state and year. Sobriety restrictions were excluded from the model.

© 2024 Furukawa NW et al. *JAMA Network Open*.

eFigure 1. Unadjusted Rate of DAA Prescriptions Fills per 100 000 Medicaid Recipients per Year by Jurisdictional Medicaid Expansion Status—US, 2014-2021

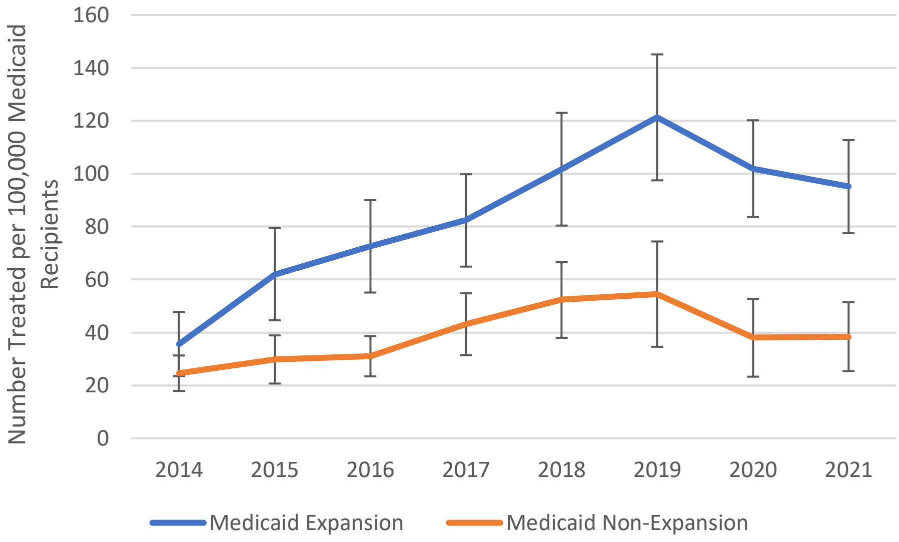

© 2024 Furukawa NW et al. *JAMA Network Open*.

eFigure 2. Unadjusted Rate of DAA Prescriptions Fills per 100 000 Medicaid Recipients per Year by Jurisdictional Fibrosis Restrictions—US, 2014-2021

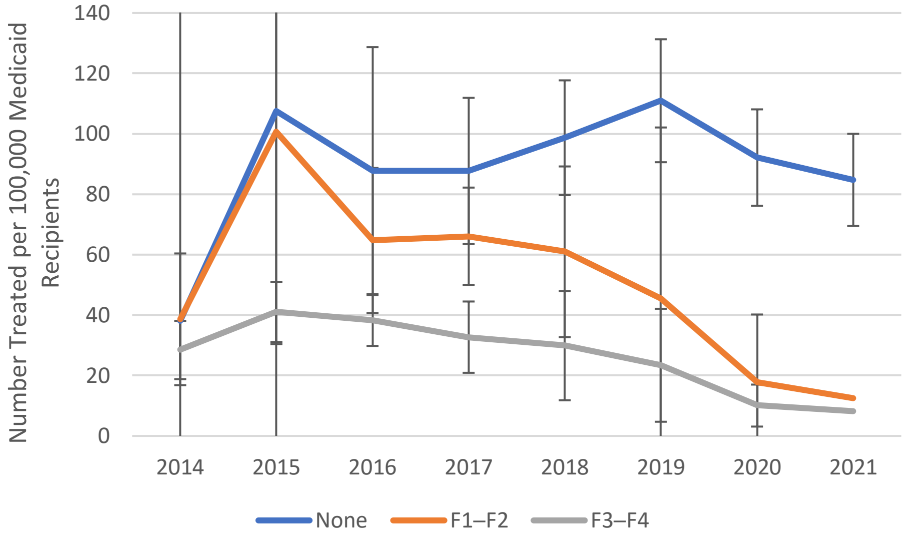

© 2024 Furukawa NW et al. *JAMA Network Open*.

eFigure 3. Unadjusted Rate of DAA Prescriptions Fills per 100 000 Medicaid Recipients per Year by Jurisdictional Sobriety Restrictions—US, 2014-2021

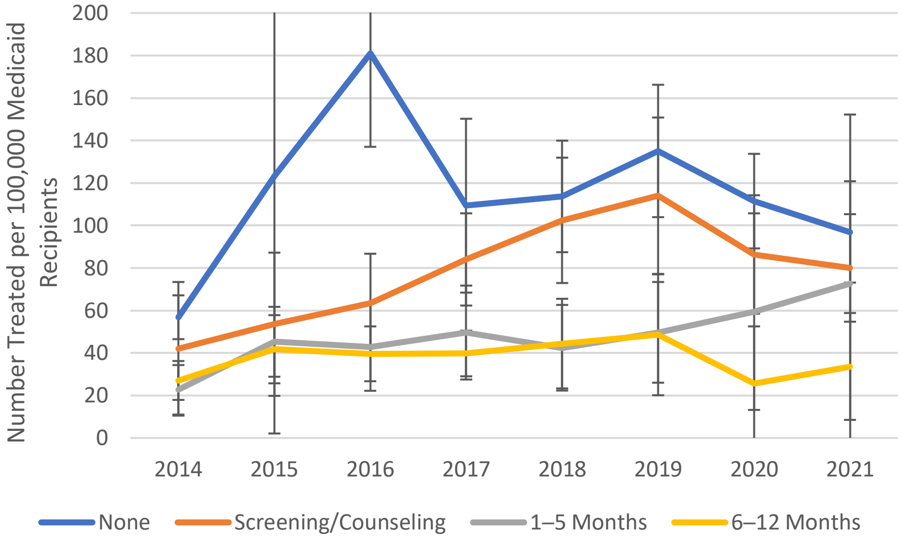

© 2024 Furukawa NW et al. *JAMA Network Open*.

eFigure 4. Unadjusted Rate of DAA Prescriptions Fills per 100 000 Medicaid Recipients per Year by Jurisdictional Prescriber Restrictions—United States, 2014-2021

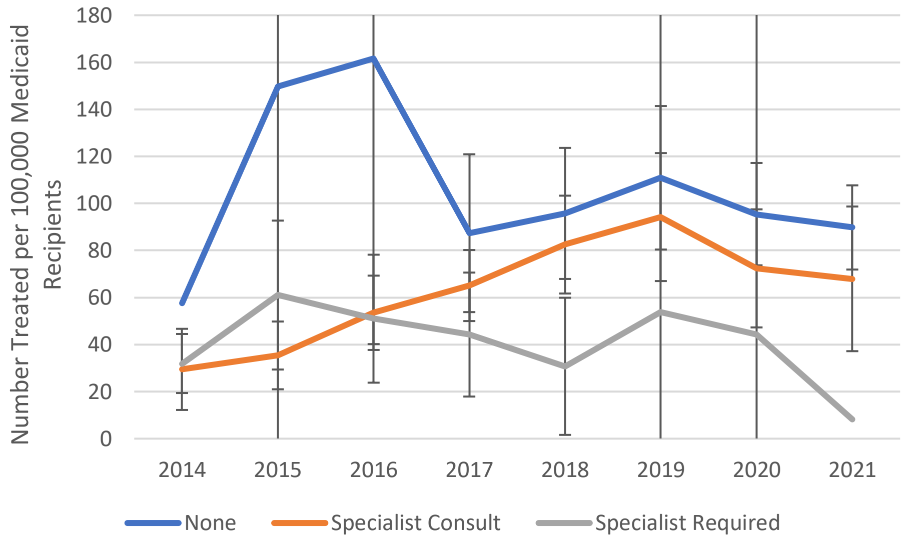

© 2024 Furukawa NW et al. *JAMA Network Open*.
