# Supplementary material for: Medicaid Expansion and Restriction Policies for Hepatitis C Treatment
Source: JAMA Netw Open. 2024 Jul 16;7(7):e2422406. doi: 10.1001/jamanetworkopen.2024.22406 (PMC11252896; doi:10.1001/jamanetworkopen.2024.22406)
Supplement: Supplement 2. — Data Sharing Statement [file jamanetwopen-e2422406-s002.pdf]

Data Sharing Statement

Furukawa. Medicaid Expansion and Restriction Policies for Hepatitis C Treatment. *JAMA Netw Open*. Published July 16, 2024. doi:10.1001/jamanetworkopen.2024.22406

Data
Data available: Yes
Data types: Other (please specify)
Additional Information: Medicaid policy assessments included. Medicaid DAA counts available for review of manuscript. CMS coding for Medicaid DAA data available upon request.
How to access data: Medicaid DAA policy hyperlink under development
When available: With publication

Supporting Documents
Document types: None

Additional Information
Who can access the data: As above
Types of analyses: As above
Mechanisms of data availability: Requests for CMS coding for Medicaid DAA counts may be made to the corresponding author.
Any additional restrictions: N/A
